# Plasmatic MicroRNAs and Treatment Outcomes of Patients with Metastatic Castration-Resistant Prostate Cancer: A Hospital-Based Cohort Study and In Silico Analysis

**DOI:** 10.3390/ijms24109101

**Published:** 2023-05-22

**Authors:** Jani Silva, Valéria Tavares, Ana Afonso, Juliana Garcia, Fátima Cerqueira, Rui Medeiros

**Affiliations:** 1 Molecular Oncology and Viral Pathology Group, Research Center of IPO Porto (CI-IPOP)/Pathology and Laboratory Medicine Department, Clinical Pathology SV/RISE@CI-IPOP (Health Research Network), Portuguese Oncology Institute of Porto (IPO Porto)/Porto Comprehensive Cancer Center (Porto.CCC), 4200-072 Porto, Portugal; valeria.tavares@ipoporto.min-saude.pt (V.T.); fatimaf@ufp.pt (F.C.); 2 AquaValor—Centro de Valorização e Transferência de Tecnologia da Água, Rua Dr. Júlio Martins, nº1, 5400-342 Chaves, Portugal; juliana.garcia@aquavalor.pt; 3 Faculty of Medicine, University of Porto (FMUP), Alameda Prof. Hernâni Monteiro, 4200-319 Porto, Portugal; 4 Abel Salazar Institute for the Biomedical Sciences (ICBAS), Universidade do Porto, Rua de Jorge Viterbo Ferreira, 228, 4050-313 Porto, Portugal; 5 Department of Oncology, Portuguese Institute of Oncology, Rua Dr. António Bernardino Almeida, 4200-072 Porto, Portugal; ana.freitas.afonso@ipoporto.min-saude.pt; 6 Centre for the Research and Technology of Agro-Environment and Biological Sciences (CITAB)/Institute for Innovation, Capacity Building and Sustainability of Agri-Food Production (Inov4Agro), University of Trás-os-Montes e Alto Douro, 5001-801 Vila Real, Portugal; 7 Instituto de Investigação, Inovação e Desenvolvimento Fernando Pessoa (FP-I3ID), Biomedical and Health Sciences (FP-BHS), Universidade Fernando Pessoa, Praça 9 de Abril, 349, 4249-004 Porto, Portugal; 8 Faculty of Health Sciences, University Fernando Pessoa, Rua Carlos da Maia, 296, 4200-150 Porto, Portugal

**Keywords:** prostatic neoplasms, abiraterone acetate, enzalutamide, prognosis, biomarkers, liquid biopsy, hsa-miR-16-5p, hsa-miR-145-5p, hsa-miR-20a-5p

## Abstract

Prostate cancer (PCa) is one of the most common malignancies among men worldwide. Inevitably, all advanced PCa patients develop metastatic castration-resistant prostate cancer (mCRPC), an aggressive phase of the disease. Treating mCRPC is challenging, and prognostic tools are needed for disease management. MicroRNA (miRNA) deregulation has been reported in PCa, constituting potential non-invasive prognostic biomarkers. As such, this study aimed to evaluate the prognostic potential of nine miRNAs in the liquid biopsies (plasma) of mCRPC patients treated with second-generation androgen receptor axis-targeted (ARAT) agents, abiraterone acetate (AbA) and enzalutamide (ENZ). Low expression levels of miR-16-5p and miR-145-5p in mCRPC patients treated with AbA were significantly associated with lower progression-free survival (PFS). The two miRNAs were the only predictors of the risk of disease progression in AbA-stratified analyses. Low miR-20a-5p levels in mCRPC patients with Gleason scores of <8 were associated with worse overall survival (OS). The transcript seems to predict the risk of death regardless of the ARAT agent. According to the in silico analyses, miR-16-5p, miR-145-5p, and miR-20a-5p seem to be implicated in several processes, namely, cell cycle, proliferation, migration, survival, metabolism, and angiogenesis, suggesting an epigenetic mechanism related to treatment outcome. These miRNAs may represent attractive prognostic tools to be used in mCRPC management, as well as a step further in the identification of new potential therapeutic targets, to use in combination with ARAT for an improved treatment outcome. Despite the promising results, real-world validation is necessary.

## 1. Introduction

Prostate cancer (PCa) represents the second most frequent malignancy and the fifth cancer-related cause of death among men worldwide, with reports of 1,414,259 new cases and 375,304 deaths in 2020 [1]. The disease is particularly common in regions with a very high human development index (HDI), though this can be, in part, explained by the rapid accessibility to prostate-specific antigen (PSA) testing, which leads to overdiagnosis and also overtreatment of PCa [2].

In general, PCa management is center stage [3]. At early disease stages, the treatment may include radical prostatectomy or radiotherapy, or even both. Observation can also be an option for localized disease management for patients at low risk of disease progression and with a life expectancy of fewer than five years [2]. For those with localized disease but with a high risk of progression and a greater life expectancy, radiotherapy can be combined with androgen deprivation therapy (ADT) [4] through orchidectomy or, more recently, through treatment with luteinizing hormone-releasing hormone (LHRH) agonists/antagonists [5]. At initial diagnosis, over 90% of PCa cases are androgen-dependent, making ADT in the reduction in circulating and tumor androgen levels and inhibition of androgen receptor (AR) signaling a good treatment option for PCa [6,7]. Inclusively, this is the standard treatment for advanced PCa. Despite an initial response, patients eventually become resistant to ADT, requiring next-generation AR inhibitors [6]. According to recent guidelines of the European Association of Urology (EAU), castration-resistant PCa (CRPC) is defined by serum levels of testosterone lower than 50 ng/dL (1.7 nmol/L) and a biochemical and/or radiological progression under effective ADT [8]. A greater understanding of CRPC due to major scientific advancements in the past two decades has identified residual androgens, ADT-induced AR splice variants, AR mutations, and growth factor signaling-mediated AR activation as common mechanisms of PCa progression toward a CRPC phenotype [5,9].

Although the vast majority of PCa patients are diagnosed at early disease stages, a subset of men develops metastatic disease after curative treatment [10]. Metastatic castration-resistant prostate cancer (mCRPC) is an inevitable and aggressive phase of prostate malignancy, with an estimated survival of three years. From a biological, metabolic, and genetic standpoint, almost all advanced PCa cases culminate in the development of a metastatic phenotype within 24 months [11,12,13]. However, in recent years, the treatment landscape has been shown to improve the outcome of mCRPC patients, particularly in terms of progression-free survival (PFS) and overall survival (OS) [5,14]. The second-generation androgen receptor axis-targeted (ARAT) agents, abiraterone acetate (AbA) and enzalutamide (ENZ), are currently approved as the first-line treatment of asymptomatic or minimally symptomatic mCRPC patients who had not received chemotherapy prior, and as the second-line treatment for those with symptomatic mCRPC who progressed after docetaxel-based chemotherapy [14,15,16,17,18].

Both AbA and ENZ target the androgen axis but through different mechanisms of action. As a derivate of pregnenolone, AbA is a selective inhibitor of androgen biosynthesis by the irreversible blockade of the cytochrome P450 (CYP) 17A1 enzyme (CYP17A1), a crucial enzyme in testosterone and estrogen synthesis in the gonad, extra-gonadal, and tumor tissues, leading to the depletion of circulating and tumoral testosterone [5,14,19]. AbA is approved in combination with low-dose prednisolone/prednisone as CYP17A1 inhibition reduces endogenous glucocorticoid synthesis [19]. Two Phase III studies, COU-AA-301 and COU-AA-302, demonstrated that AbA in mCRPC increases the PFS and OS in asymptomatic or mildly symptomatic patients and for whom chemotherapy is not yet clinically indicated, as well as adult men whose disease progresses on or after docetaxel-based chemotherapy [15,17]. ENZ, on the other hand, is an AR inhibitor that suppresses 11β-hydroxysteroid dehydrogenase-2, inhibits the binding of androgen to AR and the translocation of the activated receptor-ligand complex to the nucleus and its binding to DNA, thus competitively blocking several steps of the AR signaling pathway [5,14]. Moreover, ENZ is a potent inducer of the CYP family (e.g., CYP3A4, CYP2C19, and CYP2C9) [19]. Like AbA, two Phase III studies, namely the AFFIRM and PREVAIL trials, demonstrated that ENZ increases the PFS and OS of mCRPC patients [16,18].

Despite the substantial improvement in the treatment landscape for mCRPC, disease monitoring is not straightforward. PSA measurement per se is not a reliable biomarker at this disease phase, as visceral metastases have been observed in patients without rising PSA [13,20,21]. Henceforth, treatment optimization of mCRPC can be challenged due to a lack of validated noninvasive prognostic biomarkers.

MicroRNAs, also known as miRNAs or miRs, are small noncoding single-stranded RNAs of approximately 22 nucleotides in length with important functions in the regulation of genic expression at transcriptional and post-transcriptional levels [22,23]. The expression of over 60% of protein-coding genes is thought to be directly regulated by miRNA activity. These transcripts are recognized to carry out fundamental roles in both physiological and pathological cellular processes, including cancer development and progression. [24,25,26]. Inclusively, many have been studied as potential diagnostic and prognostic biomarkers in PCa. Beyond their role in distinguishing tumors from normal tissues, aiding in the stratification of tumors, and treatment response monitoring, miRNAs can be isolated from different body fluids, boosting their applicability in liquid biopsy [27,28,29,30].

In light of the existing evidence, this study aimed to explore the relationship between circulating miRNAs and treatment outcomes in patients with mCRPC treated with either AbA or ENZ, pre- or post-docetaxel therapy.

## 2. Results

### 2.1. miRNA Expression Levels in Plasma Samples of mCRPC, BPH, and Healthy Controls

**Figure 1:**
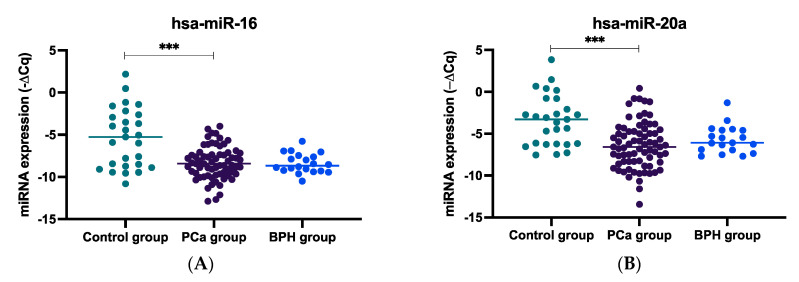

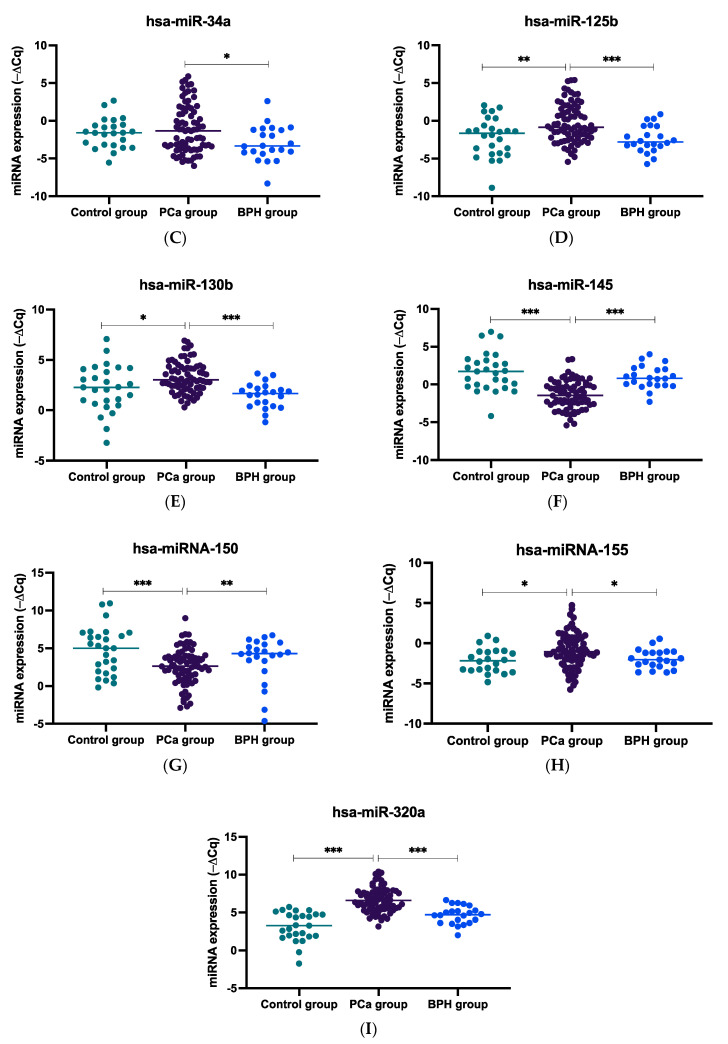
Plasmatic miRNA expression (−∆Cq) in prostate cancer (PCa) in the context of metastasis and castration resistance, benign prostate hyperplasia (BPH), and control individuals. (**A**) hsa-miR-16-5p plasmatic expression; (**B**) hsa-miR-20a-5p plasmatic expression; (**C**) hsa-miR-34a-5p plasmatic expression; (**D**) hsa-miR-125b-5p plasmatic expression; (**E**) hsa-miR-130b-3p plasmatic expression; (**F**) hsa-miR-145-5p plasmatic expression; (**G**) hsa-miR-150-5p plasmatic expression; (**H**) hsa-miR-155-5p plasmatic expression; and (**I**) hsa-miR-320a-3p plasmatic expression; * *p* < 0.05, ** *p* < 0.01, and *** *p* < 0.001.

When comparing the expression levels of the evaluated miRNAs among mCRPC, BHP, and healthy controls, circulating (plasmatic) miR-16-5p and miR-20a-5p expression levels were found to be significantly lower in PCa patients compared to the healthy control group (both, *p* < 0.001, Figure 1A, B, respectively). For miR-34a-5p, an increased expression was observed in PCa patients compared to BPH subjects (*p* = 0.018) (Figure 1C). For miR-145-5p and miR-150-5p, decreased expression levels were observed in PCa patients compared to both healthy controls and individuals with BPH (miR-145-5p, both *p* < 0.001, Figure 1F; and miR-150-5p, *p* < 0.001 and *p* = 0.003, Figure 1G). Regarding the expression of the other analyzed miRNAs, an increased expression was observed in PCa subjects compared to both healthy controls and individuals with BPH (miR-125b-5p, *p* = 0.007 and *p* < 0.001, Figure 1D; miR-130b-3p, *p* = 0.026 and *p* < 0.001, Figure 1E; miR-155-5p, *p* = 0.012 and *p* = 0.020, Figure 1H; and miR-320a-3p, both *p* < 0.001, Figure 1I).

### 2.2. Associations between the miRNA Expression and Patient Demographic and Clinicopathological Factors

The associations between the plasmatic levels of the evaluated miRNAs and the demographic and clinicopathological features of the mCRPC patients are described in Supplementary Materials, Table S1. No significant association was detected except for the one observed between age at ARAT agent initiation (<76 vs. ≥76 years) and miR-145-5p expression (*p* = 0.046). Specifically, the majority of mCRPC patients with high levels of miR-145-5p initiated therapy with an ARAT agent at a younger age (58.5%), while the opposite was observed for most of those with low levels of the transcript (30.4%). Nevertheless, according to univariable analyses, the age at ARAT agent initiation had no prognostic value in either the entire cohort or considering those treated with either AbA or ENZ (*p* > 0.05)).

### 2.3. The Impact of miRNAs on the Progression-Free Survival of mCRPC Patients

**Figure 2:**
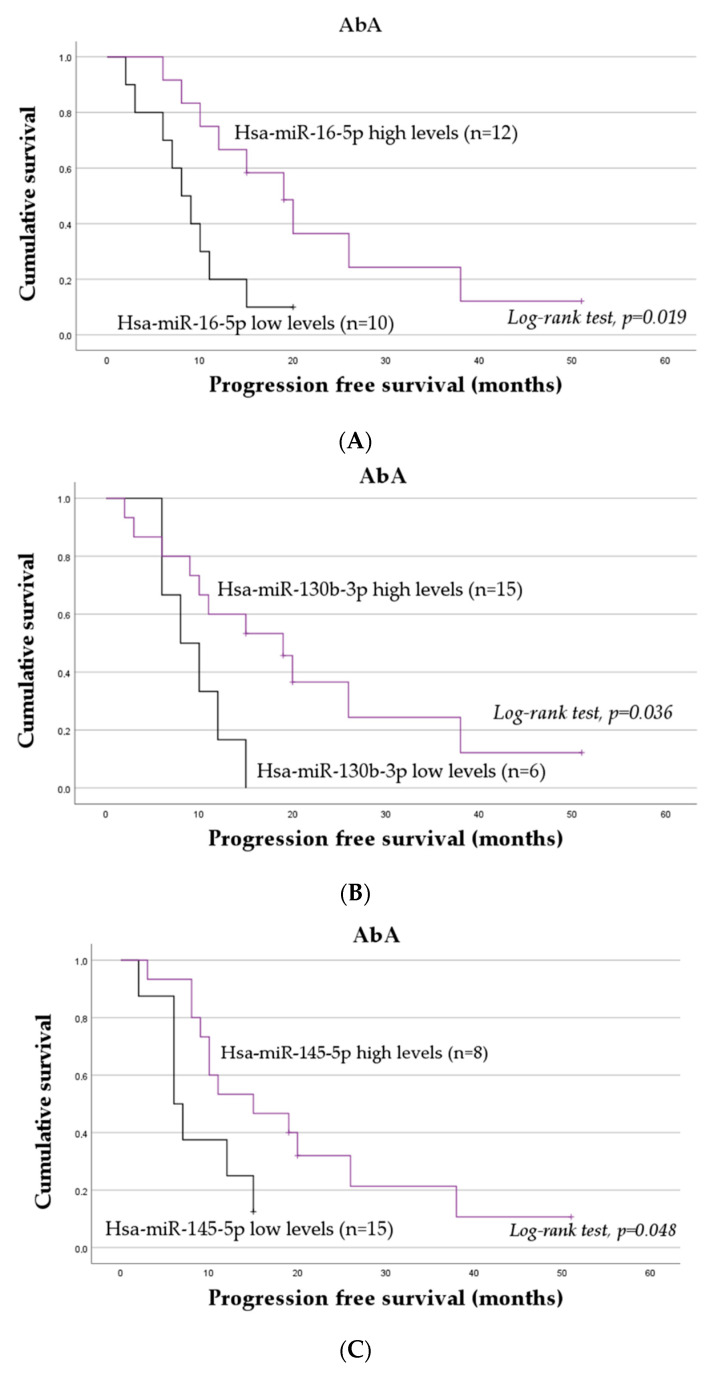

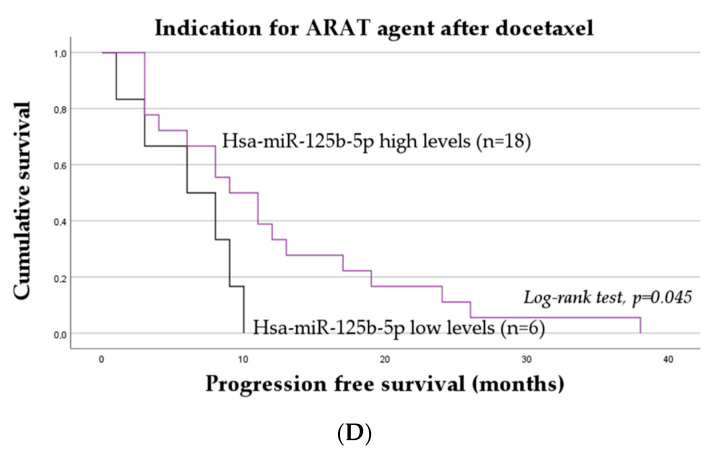
Progression-free survival of mCRPC patients according to plasmatic levels of hsa-miR-16-5p (**A**), hsa-miR-130b-3p (**B**), and hsa-miR-145-5p (**C**) for patients under abiraterone acetate (AbA) treatment, and according to plasmatic levels of hsa-miR-125b-5p (**D**) for patients with indication for ARAT agent after docetaxel-based chemotherapy.

In the overall cohort, no association between the normalized expression levels of the miRNAs and PFS was identified. In the stratified analyses, however, among mCRPC patients treated with AbA, those with low levels of miR-16-5p (Figure 2A), miR-130b-3p (Figure 2B), and miR-145-5p (Figure 2C) presented a worse PFS. Specifically, those with lower levels of miR-16-5p, miR-130b-3p, and miR-145-5p presented a mean PFS of 9.10 ± 1.62 months, 9.50 ± 1.46 months, and 8.63 ± 1.71 months, respectively, compared to a mean PFS of 22.50 ± 4.41 months, 21.03 ± 4.31 months, and 20.07 ± 3.94 months exhibited by those with high levels of these transcripts (log-rank test, *p* = 0.019, *p* = 0.036, and *p* = 0.045, respectively). According to a univariable analysis, miR-16-5p, miR-130b-3p, and miR-145-5p low levels are associated with a threefold increase in the risk of disease progression (miR-16-5p, hazard ratio (HR) = 3.13; 95% confidence interval (95% CI), 1.13–8.63; *p* = 0.028; miR-130b-3p, HR = 3.05; 95% CI, 1.00–9.33; *p* = 0.051; and miR-145-5p, HR = 2.65; 95% CI, 0.95–7.39; *p* = 0.063). To be noted, for miR-130b-3p and miR-145-5p, the association was only marginally significant.

Among mCRPC patients with an indication for ARAT after docetaxel-based chemotherapy, those with low levels of miR-125b-5p present a diminished PFS with a mean time-to-disease progression of 6.17 ± 1.45 months compared to 12.11 ± 2.26 months exhibited by those with high levels of this miRNA (log-rank test, *p* = 0.045; Figure 2D). In this subgroup, a univariable analysis showed that those with low levels of this miRNA had a threefold increase in the risk of disease progression, although the association was only marginally significant (HR = 2.63; 95% CI, 0.93–7.43; *p* = 0.069).

For the remaining miRNAs, no association with PFS was observed in the stratified analyses (*p* > 0.05).

To confirm the influence of miR-16-5p, miR-130b-3p, miR-145-5p, and miR-125b-5p on the risk of disease progression, a multivariable analysis was conducted. Demographic and clinicopathological factors with a significant impact on the risk of disease progression were first identified via a multivariable analysis using the Backward Wald selection method (Table 1). From this analysis, considering the entire cohort, the Gleason score (≥8 vs. <8), ECOG at ARAT agent initiation (2 vs. 0/1), and indication for ARAT agent (after vs. before docetaxel-based chemotherapy) were shown to be the most relevant clinical factors. Adjusting for these factors, no miRNA was found to predict the risk of disease progression in both the initial and final models after using the Backward Wald method (*p* > 0.05). In opposition, in a stratified analysis according to the ARAT agent, also adjusting for Gleason score, ECOG at ARAT agent initiation, and indication for ARAT agent, miR-16-5p and miR-145-5p were identified as the only factors with a prognostic value among patients treated with AbA. Specifically, low levels of miR-16-5p and miR-145-5p were found to be associated with a sixfold and a fivefold increase in the risk of disease progression, respectively. No associations between the transcripts’ expression levels and PFS were observed among those under ENZ in the multivariable analysis (*p* > 0.05).

### 2.4. The Impact of miRNAs on the Overall Survival of mCRPC Patients

**Figure 3:**
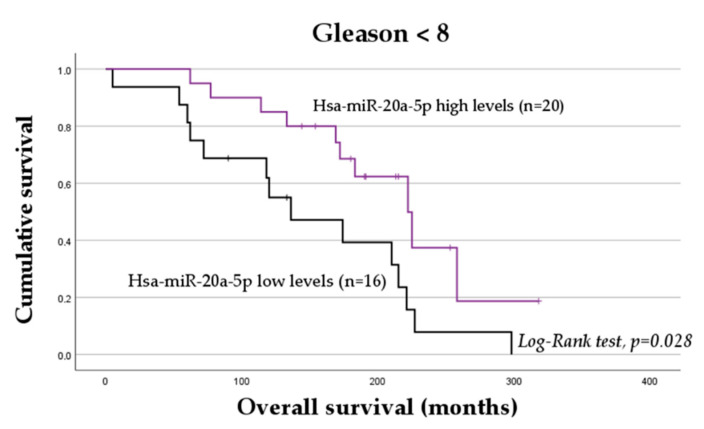
Overall survival of mCRPC patients with a Gleason score of <8 treated with either abiraterone acetate (AbA) and enzalutamide (ENZ) (n = 36) according to plasmatic levels of hsa-miR-20a-5p.

No association between the normalized expression levels of the miRNAs and OS was observed when considering the entire cohort. In the stratified analyses, however, among mCRPC patients with a Gleason score of <8, those with low levels of miR-20a-5p exhibited a worse OS (mean OS = 148.54 ± 21.78 months vs. mean OS = 213.64 ± 19.55 months; log-rank test, *p* = 0.028; Figure 3). According to the univariable analysis, among those with lower Gleason scores, miR-20a-5p low levels are associated with a twofold increase in the risk of death compared to high levels of the transcript (HR = 2.43; 95% CI, 1.07–5.50; *p* = 0.028). No significant association between the expression levels of the other transcripts and OS was observed in the stratified analyses based on the demographic and clinicopathological factors of mCRPC patients (*p* > 0.05).

To better assess the impact of miR-20a-5p on the risk of death in mCRPC patients, a multivariable analysis was conducted. Relevant demographic and clinicopathological factors significantly affecting the survival of these patients were identified in a multivariable analysis using the Backward Wald selection method (Table 2). Considering the entire cohort, patient age at PCa diagnosis (≥64 vs. <64 years), Gleason score (≥8 vs. <8), ECOG at ARAT agent initiation (2 vs. 0/1), and indication for ARAT agent (after vs. before docetaxel) were identified as prognostic factors. Adjusting for these factors, miR-20a-5p was shown to also have a prognostic value in the overall cohort. Specifically, low expression levels of miR-20a-5p were found to be associated with a twofold increase in the risk of death adjusted for the relevant demographic and clinicopathological factors. As miR-20a-5p was shown to be a relevant prognostic marker regardless of the ARAT agent, no further stratified analysis was conducted.

### 2.5. In Silico Analysis of hsa-miR-16-5p, hsa-miR-145-5p, and hsa-miR-20a-5p Targets

**Figure 4:**
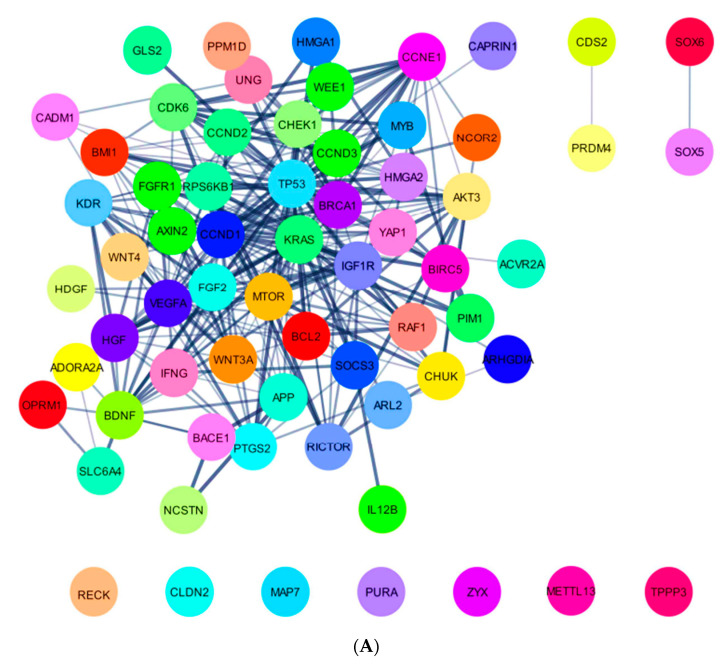

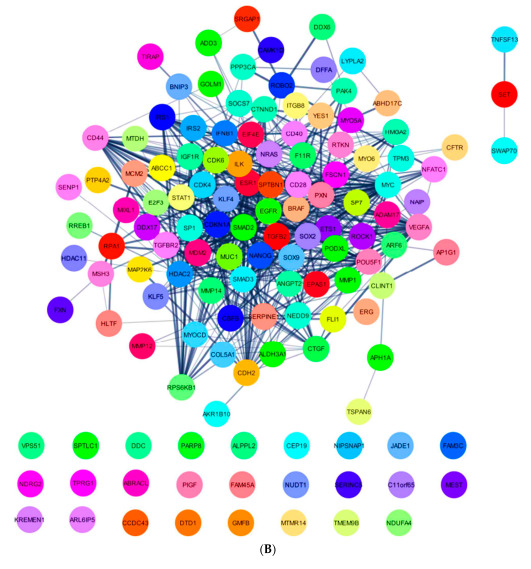

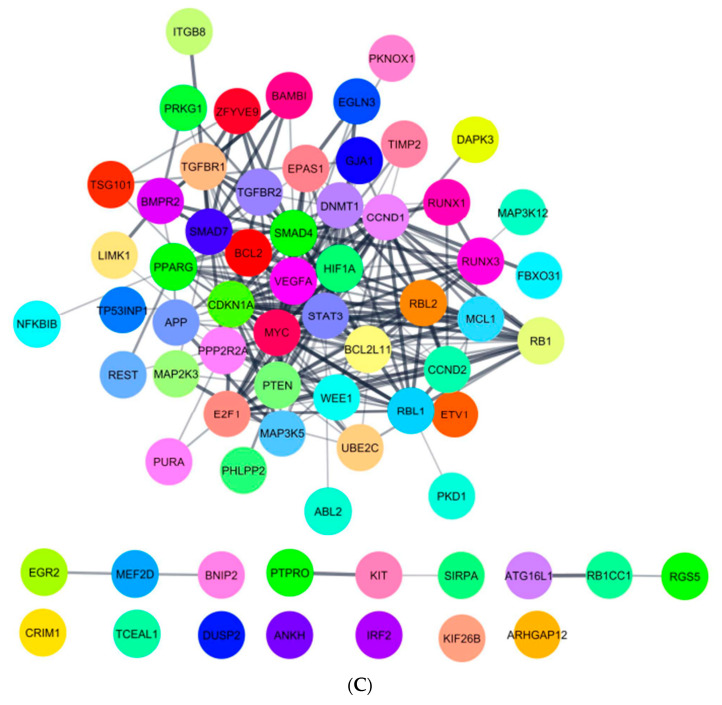
Protein–protein interaction (PPI) network of miR-16-5p (**A**), miR-145-5p (**B**), and miR-20a-5p (**C**) strong validated targets. The targets were retrieved from miRTargetLink 2.0 and the PPI was generated using the STRINGapp Protein Query from Cytoscape 3.9.1 and by applying Markov clustering (MCL).

Given the suggested role of miR-16-5p and miR-145-5p in mCRPC progression under AbA, as well as the influence of miR-20a-5p in patient survival regardless of ARAT agent, the potential biological implications of these miRNAs were further explored in in silico analyses. To do so, only strong validated targets of miR-16-5p, miR-145-5p, and miR-20a-5p were retrieved from miRTargetLink 2.0. Specifically, 66 targets were identified for miR-16-5p, 135 for miR-145-5p, and 68 for miR-20a-5p (Supplementary Materials, Table S2). Using the STRINGapp Protein Query from Cytoscape 3.9.1. and applying MCL, PPI networks were generated for miR-16-5p, miR-145-5p, and miR-20a-5p targets (miR-16-5p, 65 nodes and 298 edges, Figure 4A; miR-145-5p, 133 nodes and 578 edges, Figure 4B; and miR-20a-5p, 68 nodes and 262 edges, Figure 4C), all with a significant enrichment (*p* = 1.0 × 10^−16^).

**Figure 5:**
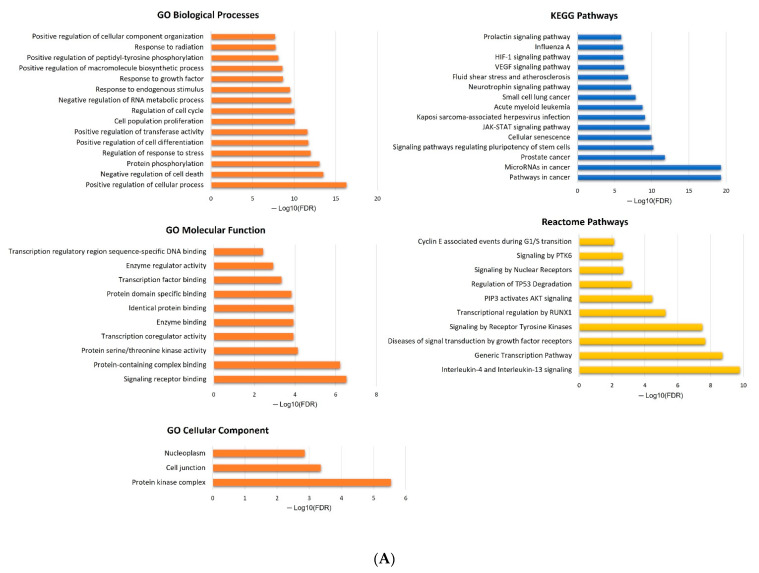

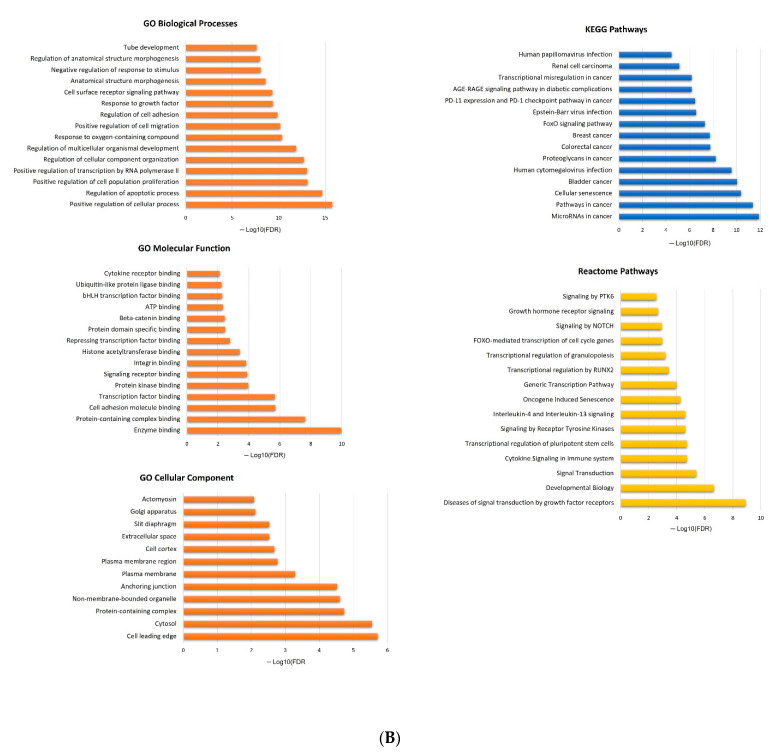

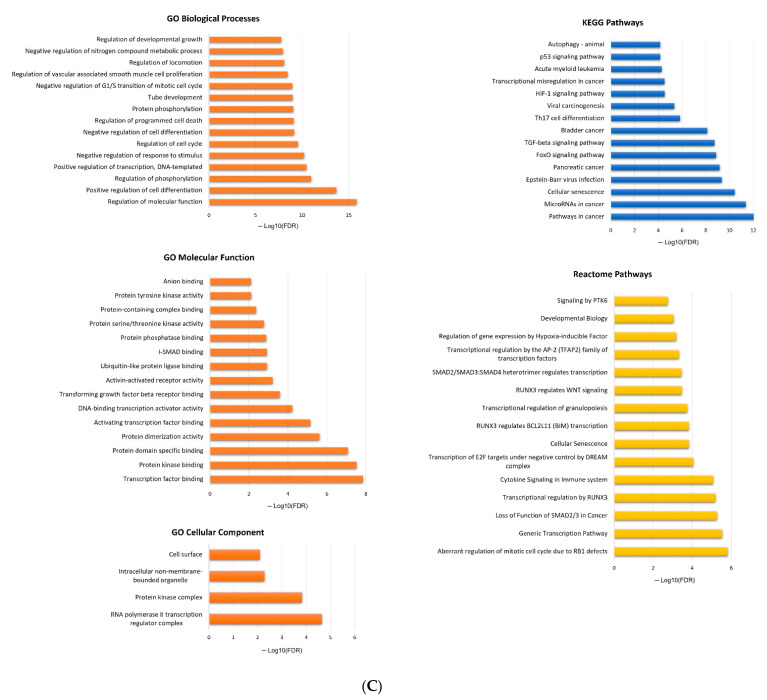
Functional enrichment analysis for hsa-miR-16-5p (**A**), hsa-miR-145-5p (**B**), and hsa-miR-20a-5p (**C**). The analysis was conducted using the STRINGGapp Protein Query from Cytoscape 3.9.1. Only the top 15 enriched terms for each miRNA were represented.

Considering the targets of each miRNA, a functional enrichment analysis was conducted leading to the identification of 333, 453, and 286 enriched terms for miR-16-5p, miR-145-5p, and miR-20a-5p, respectively. The top 15 enriched terms for GO categories, KEGG, and Reactome pathways are represented in Figure 5.

## 3. Discussion

Worldwide, prostate malignancy is a leading cause of cancer-related death [1]. Acquisition of castration resistance is an inevitable event, and to manage this condition, the identification of prognostic biomarkers and new therapeutic targets is required, a task that has been challenging [14]. Meanwhile, in an era of liquid biopsies, circulating miRNAs have attracted the attention of researchers, as these noncoding RNAs have been found to be dysregulated in a variety of diseases, including cancer [31].

In the present study, the prognostic value of nine miRNAs predicted to be implicated in PCa pathways was evaluated among mCRPC patients under treatment with the ARAT agents AbA or ENZ. The plasmatic expression levels of these miRNAs were found to be statistically different between mCRPC and healthy male individuals and/or BPH patients, suggesting a role of these transcripts in PCa susceptibility and progression. In terms of prognostic value, low plasmatic miR-16-5p and miR-145-5p expression levels were significantly associated with worse PFS among mCRPC patients under AbA treatment, which was corroborated by multivariable analyses. Additionally, low expression levels of miR-20a-5p were shown to predict a worse OS, regardless of the ARAT agent used, which was also confirmed by multivariable analyses. In opposition, although an association of miR-130b-3p and miR-125b-5p with PFS was observed among patients under AbA treatment and under ARAT post-docetaxel-based chemotherapy, respectively, the results were not confirmed in subsequent analyses.

MiR-16-5p (genomic location: 3q25.33 and 13q14.2) is a member of the miR-16 family known to be downregulated in most tumor cell lines and thus generally recognized as a tumor suppressive. Cumulative data indicate that the downregulation of miR-16-5p may promote cancer cell proliferation and survival, as well as angiogenesis and tumor metastatic dissemination, with implications in treatment responses [32]. Interestingly, miR-16-5p is the first miRNA to be confirmed as a cancer-associated gene, being frequently deleted in B-cell chronic lymphocytic leukemia [33]. Since then, several malignant diseases have been linked to this miRNA, including, but not limited to, gliomas [34], hepatocellular carcinoma [35], cervical cancer [36], gastric cancer [37], bladder cancer [38], and also PCa [39]. Like most tumor cell lines, miR-16-5p was also found to be downregulated in PCa cell lines. Surprisingly, the deletion observed in B-cell chronic lymphocytic leukemia is also reported in PCa, being the frequency associated with the tumor stage, inferring the development of more aggressive phenotypes (~30% in early stages, up to 70% in advanced stages, and ~90% in the metastatic stages) [39]. In the present study, plasmatic levels of miR-16-5p were lower in mCRPC patients compared to the levels in healthy controls, which is in line with the PCa tissue expression of this miRNA. In opposition, no significant differences were observed in the levels of mCRPC patients compared to BPH individuals. The in silico analysis suggests that this miRNA is mainly associated with cell cycle, differentiation, and death. This is in line with previous reports indicating a regulatory role of miR-16-5p in cell fate decisions involving the phenotypes senescence, apoptosis, and autophagy, through its implications in the cell cycle. Indeed, this miRNA is thought to induce DNA damage response through p53-dependent and independent pathways [32]. Inclusively, one of the potential targets of miR-16-5p is the cell cycle-related protein CCND1, a protein involved in the initiation of DNA synthesis and cell division. The miRNA is also suggested to regulate the degradation of TP53, which also didacts the cell fate decisions [40]. Another target of miR-16-5p is CDK6, a cell cycle protein upregulated in mCRPC, with roles in the AR pathway and whose inhibitors have been studied in the treatment of this condition [41]. In terms of KEGG and Reactome pathways, the in silico analysis suggests that miR-16-5p is implicated in several cancer-related pathways, including AKT, VEGF and HIF-1 pathways, among others. Inclusively, the miRNA is linked to PCa according to KEGG pathways. Concordantly, miR-16-5p is reported to have functions on resisting proliferative signaling and subduing angiogenesis, as its downregulation seems to activate the PI3K/AKT/mTOR, ANXA11/AKT and VEGFA/VEGFR1/AKT pathways to promote PCa cell proliferation [33]. Inclusively, the PI3K/AKT/mTOR pathway has been linked to the acquisition of castration resistance, and the pathway partners have been studied as potential targets in the treatment of mCRPC patients [42]. As for the HIF-1, intratumoral hypoxia mediated by its pathway is also recognized to play a key role in CRPC progression [43]. Altogether, despite the small cohort size, the present study provides experimental evidence suggesting that miR-16-5p, a tumor suppressor, could be a valuable prognostic biomarker among mCRPC patients under AbA treatment. Further investigation in larger cohorts is needed, particularly to allow more stratified analyses with sufficient statistical power.

MiR-145-5p (genomic location: 5q32) has been pinpointed as a tumor suppressor in several malignancies, including breast cancer [44], bladder cancer [45], and gastric cancer [46]. In concordance, based on the meta-analyses conducted by Zhang and Wu [47], low levels of this transcript are significantly associated with poorer outcomes, namely disease-free survival among PCa patients. Additionally, miR-145-5p downregulation in PCa tissue was shown to correlate with important prognostic variables, namely higher Gleason scores, advanced clinical stage, larger tumor diameter, and higher PSA levels, predicting a high risk of disease progression and poor patient survival [48]. In the present study, plasmatic expression levels of miR-145-5p were found to be downregulated in mCRPC patients compared to both control groups, which is in line with the reported downregulation of the miRNA in PCa tissue. Accordant to the in silico analysis, miR-145-5p seems to be particularly implicated in cell proliferation, adhesion, migration, and apoptosis, with enriched terms involving different tumor types, including bladder, breast and colorectal cancer. Inclusively, miR-145 is reported to be able to repress PCa by inhibiting cell proliferation, migration, and invasion [49]. One mechanism that has been proposed for the suppressive role of miR-145-5p in PCa progression is the inhibition of the IGF-1/1R pathway, which is associated with bone metastasis and can activate growth factor receptors, such as c-MET, a target for many inhibitors now in clinical trials for mCRPC [50,51]. miR-145 also appears to target several genes involved in PCa progression, including c-Myc, a transcription factor that regulates cell growth and differentiation, and MUC1, a transmembrane protein that promotes cell survival and invasiveness. By inhibiting the expression of these genes, miR-145 can reduce cell growth and invasion [52]. Moreover, according to the in silico analysis, miR-16-5p and miR-145-5, the two miRNAs associated with mCRPC progression in this study, seem to share targets, including VEGFA, IGF1R, and CDK6. All in all, like miR-16-5p, miR-145-5p constitutes an attractive prognostic tool for mCRPC patients under AbA treatment, which should be evaluated in additional studies with larger cohorts.

MiR-20a-5p (genomic location: 13q31.3) is a member of the miR-17-92 cluster with functions in the modulation of E2F2 and E2F3 mRNA translation in cellular processes, such as apoptosis [53,54]. Either promoting or suppressing cancer progression, the transcript has been implicated in several malignant diseases, such as breast [55], cervical [56], endometrial [57], and liver [58] cancers. Among PCa patients, plasmatic levels of miR-20a-5p were found to be downregulated, while in tumor tissue, the miRNA is reported to be overexpressed [59]. In this study, plasmatic miR-20a-5p was downregulated in mCRPC patients compared to healthy controls, which corroborates the current evidence. No significant differences were observed in the plasmatic levels of the miRNA of mCRPC patients in comparison with BPH individuals. According to the literature, miR-20a has been shown to target several genes involved in PCa progression, including TP53 and PTEN. These genes act as tumor suppressors that regulate cell growth, apoptosis, and survival, and the inhibition of their expression by miR-20a can lead to increased growth and invasion of PCa cells. Sylvestre et al. showed that translation of the E2F family of transcription factors is regulated by miR-20a, a critical process for cell cycle regulation and apoptosis [54]. In other studies, miR-20a has been found to inhibit the tumor suppressors RB1 and PTEN in DU145 cell lines [60]. Moreover, miR-20a-5p is suggested to affect the downstream pivotal signaling pathways, including PI3K/Akt [61], MAPK [62], and TGF-β [63]. Accordant to the in silico analysis, miR-20a-5p seems to be particularly implicated in cell cycle, differentiation, and apoptosis. The miRNA is also suggested to be involved in the loss of function of SMAD2/3 in cancer and has roles in several signaling pathways, including TGF-β, p53, and HIF-1 pathways, among others. TGF-β signaling is mediated through SMAD-dependent and independent pathways and has been reported to play contradictory functions in prostate tumorigenesis. Namely, this pathway may act as an inhibitor, inducing apoptosis and inhibiting proliferation in early tumor development, or as a promoter in advanced PCa, which could explain the tumor suppressive and oncogenic roles of miR-20a-5p [64]. At a molecular level, it has been proposed that SMAD4 alone, or the SMAD3/4 complex, interacts with the AR transcriptional activation domain, regulating 5-dihydrotestosterone (DHT)-induced AR transcriptional activity in PCa cell lines [64,65]. In experiments with PC3 cell lines, AR expression reduces the TGF-β1/SMAD transcriptional activity and the growth effects of TGF-β1, ultimately, preventing TGFB1-induced growth inhibition and apoptosis. On the other hand, TGF-β1 suppresses the E2F transcriptional activity of AR activation by active metabolites [64]. According to the in silico analysis, among other targets, miR-20a-5p appears to target TGFBR1, TGFBR2, SMAD4, BCL2, and E2F, suggesting a possible suppressive function in the progression of PCa. Altogether, miR-20a-5p may be a valuable prognostic biomarker to evaluate the survival of mCRPC patients.

As for the remaining miRNAs, no sustainable association (confirmed with multivariable analysis) was observed. Hence, although predicted to be implicated in linked to PCa pathways, these transcripts might not be preponderant once the castration resistance phenotype is achieved. Specifically, for miR-125b-5p, miR-130b-3p, miR-155-5p, and miR-320a-3p, their plasmatic levels were upregulated in mCRPC patients compared to both control groups, while the opposite was observed for miR-150-5p. MiR-34a-5p was upregulated in comparison with the BHP patients, but no significant differences in its plasmatic levels were observed between mCRPC patients and healthy controls. To be noted, like miR-20a-5p, plasmatic levels of miRNAs may have a different pattern of expression compared to tumor tissue. Briefly, miR-125b-5p (genomic location: 11q24.1 and 21q21.1) is reported to influence the expression of AR, the most important miRNA target in PCa. This transcript is suggested to stimulate androgen-independent growth in PCa cell lines and castrated mice PCa xenografts, likely through its antiapoptotic effects, which suggests an oncogenic role for this miRNA in PCa [66,67]. MiR-130b-3p (genomic location: 22q11.21) is reported to repress AR and MAPK signaling pathways, all in all, demonstrating its suppressive roles in PCa [68]. MiR-155-5p (genomic location: 21q21.3) gene is frequently hypermethylated in PCa; however, the same is not observed in benign prostatic tissue, which indicates a possible tumor suppressor role of this miRNA [69]. MiR-320a-3p (genomic location: 8p21.3) seems to mediate the effect of histone deacetylase inhibitors in PCa by targeting AR expression, having, apparently, a tumor-suppressive role [70]. MiR-150-5p (genomic location: 19q13.33) expression is significantly deregulated in tumor tissues, either suppressing or promoting an aggressive behavior [71,72]. In CRPC cells, this miRNA has been reported to be downregulated, imposing a tumor-suppressive role. Lastly, miR-34a-5p (genomic location: 1p36.22) belongs to the miR-34 family, whose members are shown to be downregulated in PCa. The expression of this transcript was previously found to correlate with tumor grade, advanced disease, and life expectancy in PCa patients [73]. In addition to its reported association with AR expression, miR-34a-5p seems to be a promising therapeutic option for PCa due to its known correlation with TP53 [74]. Given the biological mechanisms associated with these miRNAs, additional studies should investigate their prognostic value in the context of mCRPC.

Regarding the study limitations, the small cohort size, which may have prevented the identification of significant associations, was undeniably a major limitation. As such, the results of this preliminary study should be analyzed carefully, and additional studies are mandatory to validate these findings in a larger cohort of mCRPC patients. Furthermore, limitations associated with the in silico analyses must be recognized. In this study, the miRNAs to be evaluated were selected based on the top 100 genes predicted to be associated with PCa by STRINGapp disease Query from Cytoscape 3.9.1. Given the complex pathogenesis of PCa, and specifically mCRPC, it is possible that other genes may have a more preponderant role in mCRPC. The same can be said for the considered targets of the evaluated miRNAs. As previously mentioned, a miRNA can target a hundred mRNAs. In this study, only the strongly validated target mRNAs, meaning those with strong evidence, were taken into account. As such, the possibility of existing other target mRNAs with more relevance in mCRPC cannot be dismissed and must be investigated. The in silico analyses performed in this study can help identify biological mechanisms associated with these miRNAs and predict their potential implications in mCRPC specifically. However, for proper validation, the results of these analyses must be combined with more real-world data to provide an integrative view of the underlying mechanisms. Hence, in addition to validating the prognostic value of these miRNAs among mCRPC patients in independent and larger cohorts, functional studies exploring the roles of miR-16-5p, miR-145-5p, and miR-20a-5p in mCRPC aggressiveness are also required to better elucidate how these noncoding RNAs globally regulate the disease pathophysiologic pathways. Furthermore, the tools used for the in silico analyses have intrinsic limitations in terms of prediction given the complexity that is characteristic of biological systems and diseases such as cancer.

## 4. Materials and Methods

### 4.1. Patients, Controls, and Sample Collection

A hospital-based cohort study including 78 patients with a histopathological diagnosis of metastatic PCa and confirmed castration resistance was conducted. Patients were consecutively recruited from November 2018 to July 2019 at the Portuguese Institute of Oncology of Porto (IPOP). The median age at PCa diagnosis was 64 years (mean = 65.3 years ± 8.2 years) and the mean follow-up time was 34.2 months ± 16.6 months. All patients were under treatment with AbA or ENZ. Castration resistance was defined by blood testosterone levels <50 ng/mL under LHRH analog treatment—goserelin 10.8 mg subcutaneous q3 months [75]. Staging status at PCa diagnosis was classified according to the eighth edition of the classification system of the American Joint Committee on Cancer (AJCC) 2018 [76], and functional status was stratified according to the Eastern Cooperative Oncology Group (ECOG) [77,78]. Patients with an initial PSA of > 20 ng/mL and/or a Gleason score of ≥ 8 were considered as having an aggressive disease [79].

Healthy male blood donors recruited from 2009 to 2010 (n = 27; mean age = 60.41 ± 1.56 years) and benign prostate hyperplasia (BPH) patients recruited from 2007 to 2009 (n = 22; mean age = 67.95 ± 8.51 years; mean initial PSA = 3.47 ± 2.64 ng/mL) were included in the study for control purposes, the former as individuals without prostate pathology and the latter without prostate malignancy. Peripheral venous blood samples from patients and controls were obtained with a standard technique and collected in EDTA (ethylenediaminetetraacetic acid)-containing tubes. For mCRPC patients, the sample collection was made prior to ARAT initiation. Plasma samples were prepared by centrifugation at 3000 rpm for 5 min and subsequently stored at −80 °C until further use.

### 4.2. miRNA Selection: In Silico Analysis and Literature-Based Approach

An in silico analysis and literature review were conducted to identify the potential miRNAs implicated in PCa pathways. First, using STRINGapp disease Query from Cytoscape 3.9.1, the disease term “prostate cancer” was queried to import a full string network of the top candidate 100 disease-related genes, according to the DISEASES database. Applying the default cut-off of confidence score (i.e., 0.40), a network of 100 genes potentially associated with PCa was retrieved. Linking only those with physical interactions (meaning with a higher possibility to be indeed integrated with a network), a protein–protein interaction (PPI) was generated (72 nodes and 236 edges) with a significant enrichment (*p* = 1.0 × 10^−16^). Then, the miRNAs that most likely target these genes were further identified via miRTargetLink 2.0 (https://ccb-compute.cs.uni-saarland.de/mirtargetlink2/ (accessed on 12th January 2023). This new interactive database provides data on miRNA targets and the related pathway networks in association with other published repositories (miRDB 6.0, mirDIP 4.1, miRBase 22.1, miRPathDB 2.0, miRATBase 1.0, and miRTarBase 8.0). This database also makes use of miEAA 2.0 and GeneTrail 3.0 for functional enrichment analyses [80]. Employing the unidirectional search tool from miRTargetLink 2.0, a miRNA interaction overlap between the 72 genes was retrieved. Only strong validated miRNA-messenger RNA (mRNA) interactions, meaning confirmed experimentally through Western blot, Luciferase reporter assay, and/or qRT-PCR, were considered. Finally, based on the number of the miRNA target mRNAs (potentially involved in PCa) and on a literature review focusing on the biological functions of these miRNAs and their previously reported implications in cancer, a panel of nine miRNAs was selected: hsa-miR-16-5p, hsa-miR-20a-5p, hsa-miR-34a-5p, hsa-miR-125b-5p, hsa-miR-130b-3p, hsa-miR-145-5p, hsa-miR-150-5p, hsa-miR-155-5p, and hsa-miR-320a-3p (Table 3).

### 4.3. miRNA Extraction, cDNA Synthesis, and miRNA Relative Quantification

Plasma miRNA isolation and purification were carried out using the GRS microRNA Purification Kit (GRisP, Porto, Portugal), according to the manufacturer’s instructions. RNA concentration and purity were subsequently measured using the NanoDrop Lite spectrophotometer (Thermo Scientific^®^, Waltham, MA, USA).

MiRNA samples were the templates for complementary DNA (cDNA) synthesis using Taqman^TM^ microRNA Reverse Transcription kit (Applied Biosystems^®^, Foster City, CA, USA) and sequence-specific stem-loop primers for hsa-miR-16-5p, hsa-miR-20a-5p, hsa-miR-34a-5p, hsa-miR-125b-5p, hsa-miR-130b-3p, hsa-miR-145-5p, hsa-miR-150-5p, hsa-miR-155-5p, hsa-miR-320a-3p, U6 snRNA, RNU-44, RNU-48, and RNU6b, according to the manufacturer’s protocols.

MiRNA expression levels among mCRPC, BHP, and healthy controls were analyzed using quantitative Real-Time PCR (RT-qPCR) in StepOneTMqPCR Real-Time equipment. Each reaction was conducted using 5 µL of 2 × TaqMan^®^ Fast Advanced Master Mix (Thermo Fisher Scientific^®^), 3.0 µL of nuclease-free water, 0.5 µL of 20 × specific TaqMan^TM^ microRNA assays for hsa-miR-16-5p (Assay ID 000391, Applied Biosystems), hsa-miR-20a-5p (Assay ID 000580, Applied Biosystems), hsa-miR-34a-5p (Assay ID 000426, Applied Biosystems), hsa-miR-125b-5p (Assay ID 000449, Applied Biosystems), hsa-miR-130b-3p (Assay ID 000456, Applied Biosystems), hsa-miR-145-5p (Assay ID 002278, Applied Biosystems), hsa-miR-150-5p (Assay ID 000473, Applied Biosystems), hsa-miR-155-5p (Assay ID 002623, Applied Biosystems), hsa-miR-320a-3p (Assay ID 002277, Applied Biosystems), U6 snRNA (Assay ID 001973, Applied Biosystems), RNU-44 (Assay ID 001094, Applied Biosystems), RNU-48 (Assay ID 001006, Applied Biosystems), and RNU6b (Assay ID 001093, Applied Biosystems), and 1.5 µL of cDNA samples in a total volume of 10 µL. As measures of quality control, negative controls (without cDNA) were included in each PCR run, and the quantification was performed in triplicates for each sample (Ct standard deviation superior to 0.5 were dismissed). Likewise, the endogenous controls were amplified in all PCR runs for all of the analyzed samples. Thermal cycling conditions were described previously [97]. Data analysis was performed using StepOneTM v2.2 Software (Applied Biosystems) with the same baseline and threshold set for each plate to generate quantification cycle (Cq) values for all of the miRNAs in each sample.

### 4.4. Statistical Analysis

Statistical analysis was performed using IBM SPSS Statistics software for Windows (Version 27.0) and GraphPad Prism 9.0 software for Windows (GraphPad Software Inc., La Jolla, CA, USA). The quality of the endogenous controls was analyzed using the BestKeeper software [98]. Among the tested endogenous controls, U6 snRNA was the one selected to normalize the miRNA expression levels given its stable expression. The miRNA relative expression was determined using the Livak method (2^−∆∆Ct^ method).

Outliers of the normalized expression levels of the transcripts were identified using the interquartile range (IQR) method and subsequently dismissed for further analyses. The Kolmogorov–Smirnov test was applied to test where the normalized miRNA expression levels were normally distributed. Depending on the distribution, the Student’s *t*-test (normal distribution) or Mann–Whitney U test (not normal distribution) were employed to assess the statistical differences in miRNA expression levels in the three groups of our cohort.

Two profiles of expression for each miRNA were set (low versus high expression levels) based on the median value of expression level. The associations between the miRNA expressions and the patient demographic and clinicopathological factors were assessed using the Chi-square test. Patient demographic and clinicopathological factors included: patient age at disease diagnosis (≥64 vs. <64 years), metastasis at disease diagnosis (yes vs. no), PSA at disease diagnosis (>20 vs. ≤20 ng/mL), Gleason score (≥8 vs. <8), patient age at ARAT agent initiation (≥76 vs. <76 years), ECOG at ARAT agent initiation (2 vs. 0/1), indication for ARAT agent (after vs. before docetaxel-based chemotherapy), and ARAT agent (ENZ vs. AbA). The variable categories of patient age at disease diagnosis and upon ARAT agent initiation were defined based on the median values of these factors among our cohort.

Measures of clinical outcome were PFS and OS, which were described in detail previously [75]. Kaplan–Meier and Log-Rank tests were employed to evaluate the impact of the miRNA expression levels on PFS and OS in both the entire cohort and according to patient demographic and clinicopathological factors. For those significantly associated with PFS or OS, or both, the risk of disease progression and/or the risk of death in the entire cohort or according to ARAT was estimated employing the Cox proportional hazards model adjusted for the relevant demographic and clinicopathological factors previously identified using the Backward Wald method.

For all analyses, a level of significance of 5% was established.

### 4.5. In Silico Analyses

For miRNAs associated with the risk of disease progression and/or the risk of death, their potential biological implications were further explored by conducting more in-depth in silico analyses. To do so, the miRTargetLink 2.0. database was employed to identify all of the microRNA targets. To be noted, a microRNA may have several hundred targets and a single mRNA can be targeted by several miRNAs [99]. In this study, only strong validated targets of the transcripts were considered, without restriction to PCa pathways. After identifying the miRNA targets, the STRINGapp Protein Query from Cytoscape 3.9.1 was employed to generate a full string network of PPI for each miRNA. Markov clustering (MCL) was further applied to cluster the proteins based on their STRING interaction score. Lastly, a functional enrichment analysis was conducted considering a false discovery rate (FDR) of < 0.01 and eliminating the redundant terms (cut-off of 0.5). The top 15 enriched terms for GO categories, KEGG, and Reactome pathways were illustrated for each miRNA.

## 5. Conclusions and Future Perspectives

Among men, PCa represents one of the most diagnosed cancers worldwide. Although most patients present early disease stages at diagnosis, a subset of them eventually develop mCRPC, which is an aggressive phase of the malignant disease. Circulating miRNAs may improve the clinical prediction of mCRPC prognosis and enhance patient quality of life. Findings of the present study pinpoint plasmatic miR-16-5p and miR-145-5p as predictors of disease progression under AbA treatment, while plasmatic miR-20a-5p seems to predict the survival of mCRPC patients regardless of the ARAT used. In silico analyses and the existing literature indicate several targets of these miRNAs, which seem to be involved in AR-related pathways and are currently being investigated as therapeutic targets for mCRPC patients under ARAT.

Given the potential clinical benefits, further investigation on the predictive and prognostic value of miR-16-5p, miR-145-5p, and miR-20a-5p among mCRPC patients is required. Inclusively, their expression levels in PCa tissues need to be better evaluated to assess their suppressive and/or oncogenic functions. Furthermore, the role of these transcripts in the context of hormone sensitivity also needs to be investigated given the implications for patient management. Despite the promising results concerning miR-16-5p, miR-145-5p, and miR-20a-5p, these transcripts can have up to several hundred targets, and a single mRNA can be targeted by several miRNAs [99]. Thus, more real-world studies providing functional data on these miRNAs are necessary to better dissect the underlying biological mechanisms in mCRPC. The goal is to design a panel of relevant miRNAs in mCRPC that may help clinicians to assess patient prognosis, as well as identify new potential therapeutic targets to use in combination with ARAT for a better treatment outcome.

## Figures and Tables

**Table 1:** Multivariable analysis using the Backward Wald method on the risk of disease progression of mCRPC patients considering patient demographic and clinicopathological factors and plasmatic levels of miRNAs.

Factor	aHR (95% CI)	*p*-Value	aHR (95% CI)	*p*-Value
**Entire cohort (n = 55)**	**Initial model**		Final model **	
Age at disease diagnosis (≥64 vs. <64 years *)	1.59 (0.75–3.37)	0.229	-	-
Metastasis at disease diagnosis (Yes vs. no *)	0.65 (0.30–1.38)	0.259	-	-
PSA at disease diagnosis (>20 vs. ≤20 ng/mL *)	0.66 (0.30–1.43)	0.292	-	-
Gleason score (≥8 vs. <8 *)	2.60 (1.14–5.95)	**0.024**	1.75 (0.94–3.27)	0.080
Age at ARAT initiation (≥76 vs. <76 years *)	0.75 (0.33–1.72)	0.496	-	-
ECOG at ARAT agent initiation (2 vs. 0/1 *)	6.48 (1.94–21.62)	**0.002**	4.09 (1.30–12.87)	**0.016**
Indication for ARAT agent (After vs. before docetaxel *)	3.15 (1.59–6.25)	**<0.001**	2.93 (1.51–5.69)	**0.001**
ARAT agent (ENZ vs. AbA *)	0.74 (0.36–1.54)	0.425	-	-
**Patients under AbA (n = 19)**	**Initial model**		Final model **	
Gleason score (≥8 vs. <8 *)	1.65 (0.45–6.12)	0.451	-	-
ECOG at ARAT agent initiation (2 vs. 0/1 *)	NA	NA	-	-
Indication for ARAT agent (After vs. before docetaxel *)	1.00 (0.28–3.57)	0.995	-	-
Hsa-miR-16-5p (Low vs. high levels *)	8.99 (2.09–38.71)	**0.003**	5.58 (1.54–20.28)	**0.009**
Hsa-miR-125b-5p (Low vs. high levels *)	0.32 (0.07–1.45)	0.140	-	-
Hsa-miR-130b-3p (Low vs. high levels *)	4.09 (0.46–36.79)	0.208	-	-
Hsa-miR-145-5p (Low vs. high levels *)	3.36 (0.40–28.52)	0.267	4.71 (1.18–18.88)	**0.029**

Bold values were regarded as statistically significant. * Reference group; ** Final model after applying the Backward Wald selection method. AbA, abiraterone acetate; aHR, adjusted hazard ratio; ARAT, androgen receptor-axis-targeted therapies; CI, confidence interval; ECOG, Eastern Cooperative Oncology Group; ENZ, enzalutamide; mCRPC, metastatic castration-resistant prostate cancer; NA, not applicable due to the insufficient number of patients with ECOG 2; and PSA, prostate-specific antigen.

**Table 2:** Multivariable analysis using the Backward Wald method on the risk of death of mCRPC patients considering patient demographic and clinicopathological factors and plasmatic levels of hsa-miR-20a-5p.

Factor	aHR (95% CI)	*p*-Value	aHR (95% CI)	*p*-Value
**Entire cohort (n = 64)**	**Initial model**		Final model **	
Age at disease diagnosis (≥64 vs. <64 years *)	8.82 (3.11–25.01)	**<0.001**	7.56 (3.18–18.01)	**<0.001**
Metastasis at disease diagnosis (Yes vs. no *)	2.24 (0.79–6.34)	0.128	-	-
PSA at disease diagnosis (>20 vs. ≤20 ng/mL *)	0.59 (0.25–1.37)	0.217	-	-
Gleason score (≥8 vs. <8 *)	2.76 (1.21–6.29)	**0.016**	2.66 (1.33–5.32)	**0.006**
Age at ARAT initiation (≥76 vs. <76 year s *)	0.77 (0.34–1.77)	0.537	-	-
ECOG at ARAT agent initiation (2 vs. 0/1 *)	5.81 (1.35–25.01)	**0.018**	5.73 (1.47–22.28)	**0.012**
Indication for ARAT agent (After vs. before docetaxel *)	4.20 (1.87–9.46)	**<0.001**	4.50 (2.03–9.98)	**<0.001**
ARAT agent (ENZ vs. AbA *)	0.94 (0.45–1.99)	0.873	-	-
**Entire cohort (n = 70)**	**Initial model**		Final model **	
Age at disease diagnosis (≥64 vs. <64 years *)	5.70 (2.60–12.50)	**<0.001**	NA	NA
Gleason score (≥8 vs. <8 *)	2.49 (1.29–4.78)	**0.006**	NA	NA
ECOG at ARAT agent initiation (2 vs. 0/1 *)	9.37 (2.35–37.43)	**0.002**	NA	NA
Indication for ARAT agent (After vs. before Docetaxel *)	4.89 (2.32–10.30)	**<0.001**	NA	NA
Hsa-miR-20a-5p (Low vs. high levels)	2.48 (1.28–4.83)	**0.007**	NA	NA

Bold values were regarded as statistically significant. * Reference group; ** Final model after applying the Backward Wald selection method. AbA, abiraterone acetate; aHR, adjusted hazard ratio; ARAT, androgen receptor-axis-targeted therapies; CI, confidence interval; ECOG, Eastern Cooperative Oncology Group; ENZ, enzalutamide; mCRPC, metastatic castration-resistant prostate cancer; NA, not applicable as the initial model does not differ from the final model; and PSA, prostate-specific antigen.

**Table 3:** Selected miRNAs targeting proteins implicated in prostate cancer.

MiRNA	MiRNA Genomic Location	Potential Targets	Source	References
Hsa-miR-16-5p	3q25.33 13q14.2	CCND1	MIRT001225	[81,82,83]
		BCL2	MIRT001800	
		VEGFA	MIRT003890	
		TP53	MIRT005764	
Hsa-miR-20a-5p	13q31.3	CCND1	MIRT000179	[84,85]
		BCL2	MIRT003011	
		PTEN	MIRT003369	
		VEGFA	MIRT004450	
		MYC	MIRT005289	
		STAT3	MIRT050559	
Hsa-miR-34a-5p	1p36.22	MYC	MIRT000695	[73,86,87,88]
		CCND1	MIRT001013	
		BCL2	MIRT002298	
		TP53	MIRT007112	
		AKT1	MIRT733152	
Hsa-miR-125b-5p	11q24.1 21q21.1	TP53	MIRT000535	[66,67,89]
		AKT1	MIRT004363	
		STAT3	MIRT005006	
		BCL2	MIRT006253	
		EGFR	MIRT733343	
Hsa-miR-130b-3p	22q11.21	STAT3	MIRT053071	[68,90,91,92]
		PTEN	MIRT054460	
Hsa-miR-145-5p	5q32	EGFR	MIRT003325	[48,49,93]
		MYC	MIRT004290	
		VEGFA	MIRT006215	
Hsa-miR-150-5p	19q13.33	VEGFA	MIRT004272	[71,72,94,95]
		TP53	MIRT052652	
Hsa-miR-155-5p	21q21.3	CCND1	MIRT020946	[69,96]
		MYC	MIRT054028	
		PTEN	MIRT734893	
Hsa-miR-320a	8p21.3	MYC	MIRT044759	[70,94]
		PTEN	MIRT438486	
		VEGFA	MIRT732583	

Genomic location according to Ensembl (https://www.ensembl.org/index.html (accessed on 15 January 2023)). For some miRNA–mRNA interactions, the validation was performed by multiple experiments and only one for each mRNA–miRNA interaction is represented in the table.

## Data Availability

The data presented in this study are available upon request from the corresponding authors. The data are not publicly available due to the privacy of participating patients and the hospital.

## Supplementary Materials

The following supporting information can be downloaded at: https://www.mdpi.com/article/10.3390/ijms24109101/s1.
