# Supplementary material for: Plasmatic MicroRNAs and Treatment Outcomes of Patients with Metastatic Castration-Resistant Prostate Cancer: A Hospital-Based Cohort Study and In Silico Analysis
Source: Int J Mol Sci. 2023 May 22;24(10):9101. doi: 10.3390/ijms24109101 (PMC10219330; doi:10.3390/ijms24109101)
Supplement: Supplementary file 1 [file ijms-24-09101-s001.zip › ijms-2363707-supplementary.pdf]

International Journal of *Molecular Sciences*

MDPI

Supplementary Materials

Table S1. Association of miRNAs expression levels with demographic and clinicopathological factors of mCRPC patients.

Demographic or clinicopathological factors	Hsa-miR-16-5p N	Low levels	High levels	*p*-Value	Hsa-miR-20a-5p N	Low levels	High levels	*p*-Value	Hsa-miR-34a-5p N	Low levels	High levels	*p*-Value
**Age at disease diagnosis, years**												
<64	32	14	18	0.966	36	15	21	0.975	37	15	22	0.252
>=64	36	17	19		38	17	21		41	23	18	
**Initial PSA, ng/mL**												
<=20	32	15	17	1.000	35	18	17	0.456	37	18	19	0.770
>20	24	11	13		26	10	16		27	15	22	
**Gleason**												
<8	35	18	17	0.502	36	16	20	0.972	36	20	16	0.355
>=8	30	12	18		34	14	20		38	16	22	
**Metastasis at diagnosis**												
No	51	24	27	0.888	56	24	32	1.000	58	30	28	0.519
Yes	17	7	10		18	8	10		20	8	12	
**Indication for ARAT agent**												
Before docetaxel	43	22	21	0.338	48	24	24	0.178	52	26	26	0.936
After docetaxel	25	9	16		26	8	18		26	12	14	
**Age at ARAT agent initiation**												
<76	34	12	22	0.144	37	15	22	0.814	39	15	24	0.113
>=76	34	19	15		37	17	20		39	23	16	
**ARAT agent**												
AbA	26	12	14	1.000	27	13	14	0.688	28	16	12	0.380
ENZ	42	19	23		47	19	28		50	22	28	
**ECOG at ARAT agent initiation**												
0 and 1	64	29	35	1.000	68	30	38	0.935	72	33	39	0.180
2	4	2	2		6	2	4		6	5	1	

	Hsa-miR-125b-5p N	Low levels	High levels	*p*-Value	Hsa-miR-130b-3p N	Low levels	High levels	*p*-Value	Hsa-miR-145-5p N	Low levels	High levels	*p*-Value
**Age at disease diagnosis, years**												
<64	37	14	23	0.924	32	11	21	0.978	35	9	26	0.584
>=64	41	17	24		40	15	25		41	14	27	
**Initial PSA, ng/mL**												
<=20	37	15	22	0.726	33	15	18	0.196	36	11	25	1.000
>20	27	13	14		27	7	20		27	8	19	
**Gleason**												
<8	36	16	20	0.507	34	12	22	0.877	36	10	26	0.499
>=8	38	13	25		35	14	21		37	13	24	
**Metastasis at diagnosis**												
No	58	23	35	1.000	52	20	32	0.692	56	17	39	1.000
Yes	20	8	12		20	6	14		20	6	14	
**Indication for ARAT agent**												
Before docetaxel	52	25	27	0.060	48	18	30	0.931	50	17	33	0.471
After docetaxel	26	6	20		24	8	16		26	6	20	

**Age at ARAT agent initiation**												
<76	39	14	25	0.644	35	13	22	1.000	38	7	31	**0.046**
>=76	39	17	22		37	13	24		38	16	22	
**ARAT agent**												
AbA	28	12	16	0.858	25	8	17	0.786	27	10	17	0.488
ENZ	50	19	31		47	18	29		49	13	36	
**ECOG at ARAT agent initiation**												
0 and 1	72	26	46	0.066	68	26	42	0.312	71	23	48	0.308
2	6	5	1		4	0	4		5	0	5	
	**Hsa-miR-150-5p**				**Hsa-miR-155-5p**				**Hsa-miR-320a-3p**			
	**N**	**Low levels**	**High levels**	***p*-Value**	**N**	**Low levels**	**High levels**	***p*-Value**	**N**	**Low levels**	**High levels**	***p*-Value**
**Age at disease diagnosis, years**												
<64	36	19	17	0.254	35	16	19	0.693	34	8	26	0.902
>=64	38	26	12		39	15	24		40	11	29	
**Initial PSA, ng/mL**												
<=20	35	23	12	0.501	35	18	17	0.540	35	9	26	0.790
>20	26	14	12		25	10	15		27	9	18	
**Gleason**												
<8	34	20	14	0.850	36	16	20	0.972	35	9	26	1.000
>=8	36	23	13		34	14	20		36	10	26	
**Metastasis at diagnosis**												
No	55	33	22	1.000	55	25	30	0.431	54	14	40	1.000
Yes	19	12	7		19	6	13		20	5	15	
**Indication for ARAT agent**												
Before docetaxel	48	29	19	1.000	49	22	27	0.628	49	13	36	1.000
After docetaxel	26	16	10		25	9	16		25	6	19	
**Age at ARAT agent initiation**												
<76	38	19	19	0.086	36	14	22	0.784	37	9	28	1.000
>=76	36	26	10		38	17	21		37	10	27	
**ARAT agent**												
AbA	25	18	7	0.247	28	12	16	1.000	25	7	18	0.964
ENZ	49	27	22		46	19	27		49	12	37	
**ECOG at ARAT agent initiation**												
0 and 1	68	40	28	0.458	69	29	40	1.000	69	18	51	1.000
2	6	5	1		5	2	3		5	1	4	

AbA, Abiraterone acetate; ARAT, Second-generation androgen receptor axis-targeted agent; ECOG, Eastern Cooperative Oncology Group; ENZ, Enzalutamide; PSA, Prostate- specific antigen.

Table S2. Strong validated microRNA targets retrieved from miRTargetLink 2.0 database.

Hsa-miR-16-5p		Hsa-miR-145-5p		Hsa-miR-20a-5p	
**Target**	**Source**	**Target**	**Source**	**Target**	**Source**
BMI1	MIRT000266	ABCC1	MIRT706010	HIF1A	MIRT000002
HMGA1	MIRT000270	ABHD17C	MIRT021539	TCEAL1	MIRT000178
ACVR2A	MIRT000536	ABRACL	MIRT021534	**CCND1**	MIRT000179
**CDK6**	MIRT000939	ADAM17	MIRT007275	E2F1	MIRT000180
CCND3	MIRT000940	ADD3	MIRT437436	BMPR2	MIRT000181
PURA	MIRT001425	AKR1B10	MIRT021503	**CDKN1A**	MIRT000597
**CCND1**	MIRT001225	ALDH3A1	MIRT021523	**TGFBR2**	MIRT001785
CCNE1	MIRT001226	ALPPL2	MIRT021509	RUNX1	MIRT003742
FGF2	MIRT001468	ANGPT2	MIRT438013	MAP3K12	MIRT003010
PTGS2	MIRT001426	AP1G1	MIRT021529	BCL2	MIRT003011
ARL2	MIRT001478	APH1A	MIRT021538	MEF2D	MIRT003012
ARHGDIA	MIRT001479	ARF6	MIRT278608	PTEN	MIRT003369
BCL2	MIRT001800	ARL6IP5	MIRT021502	APP	MIRT003382
WNT3A	MIRT003430	BNIP3	MIRT000305	**VEGFA**	MIRT004450
BRCA1	MIRT003328	BRAF	MIRT053219	BCL2L11	MIRT004570
AKT3	MIRT003329	C11orf65	MIRT021504	MYC	MIRT005289
**CCND2**	MIRT003431	CAMK1D	MIRT733960	IRF2	MIRT006178
CADM1	MIRT004382	CBFB	MIRT000676	BNIP2	MIRT005481
**VEGFA**	MIRT003890	CCDC43	MIRT021517	SMAD4	MIRT005631
MYB	MIRT004637	CD28	MIRT438542	**CCND2**	MIRT005854
TPPP3	MIRT004470	CD40	MIRT734755	RB1	MIRT005857
CAPRIN1	MIRT005086	CD44	MIRT053216	RBL1	MIRT005858
PPM1D	MIRT005360	CDH2	MIRT007288	RBL2	MIRT005859
KDR	MIRT006794	CDK4	MIRT005879	WEE1	MIRT005860
CHUK	MIRT005554	**CDK6**	MIRT054379	KIT	MIRT006180
TP53	MIRT005764	CDKN1A	MIRT000575	PPARG	MIRT006754
ZYX	MIRT006534	CEP19	MIRT021514	EGLN3	MIRT006289
NCOR2	MIRT006651	CFTR	MIRT438886	BAMBI	MIRT006755
AXIN2	MIRT006751	CLINT1	MIRT000678	CRIM1	MIRT006756
FGFR1	MIRT006795	COL5A1	MIRT053211	MAP2K3	MIRT006772
**HMGA2**	MIRT006804	CRNDE	MIRT734326	PURA	MIRT007002
PIM1	MIRT006870	CTGF	MIRT007247	SIRPA	MIRT035531
UNG	MIRT007301	CTNND1	MIRT053657	ARHGAP12	MIRT031082
IFNG	MIRT006912	DDC	MIRT437834	TSG101	MIRT031083
**RPS6KB1**	MIRT031441	DDX17	MIRT006903	STAT3	MIRT050559
RECK	MIRT031373	DDX6	MIRT241310	LIMK1	MIRT052914
PRDM4	MIRT031524	DFFA	MIRT004580	UBE2C	MIRT050549
MTOR	MIRT031485	DTD1	MIRT021511	GJA1	MIRT053007
MAP7	MIRT031639	E2F3	MIRT438350	PHLPP2	MIRT052971
WEE1	MIRT031934	EGFR	MIRT003325	DUSP2	MIRT053023
APP	MIRT031838	EIF4E	MIRT005878	MAP3K5	MIRT053208
SOX6	MIRT052901	EPAS1	MIRT053030	ITGB8	MIRT053109
CDS2	MIRT032053	ERG	MIRT007094	EGR2	MIRT053563
RAF1	MIRT053089	ESR1	MIRT006774	SMAD7	MIRT053159
KRAS	MIRT053090	ETS1	MIRT053038	TP53INP1	MIRT053505
SLC6A4	MIRT053620	F11R	MIRT021501	ABL2	MIRT054860

**IGF1R**	MIRT053088	FAM3C	MIRT021525	MCL1	MIRT053332
BDNF	MIRT437463	FAM45A	MIRT021527	ZFYVE9	MIRT071903
YAP1	MIRT054284	FLI1	MIRT004496	REST	MIRT213203
SOX5	MIRT054896	FSCN1	MIRT003543	ATG16L1	MIRT087604
BIRC5	MIRT054909	FXN	MIRT734230	ANKH	MIRT095719
CHEK1	MIRT265077	GMFB	MIRT021506	PRKG1	MIRT437765
RICTOR	MIRT437349	GOLM1	MIRT021516	ETV1	MIRT438054
HDGF	MIRT438686	HDAC11	MIRT438718	RGS5	MIRT437944
BACE1	MIRT732050	HDAC2	MIRT007307	FBXO31	MIRT438351
WNT4	MIRT438738	HLTF	MIRT021505	DNMT1	MIRT438806
NCSTN	MIRT732051	**HMGA2**	MIRT437833	EPAS1	MIRT438160
IL12B	MIRT732755	IFNB1	MIRT004616	PKD1	MIRT438812
OPRM1	MIRT732142	**IGF1R**	MIRT004931	RUNX3	MIRT561650
ADORA2A	MIRT733031	ILK	MIRT007180	PKNOX1	MIRT513751
CLDN2	MIRT735443	IRS1	MIRT000731	KIF26B	MIRT732249
HGF	MIRT733537	IRS2	MIRT006332	RB1CC1	MIRT731268
SOCS3	MIRT733215	ITGB8	MIRT006494	NFKBIB	MIRT731837
METTL13	MIRT733916	JADE1	MIRT021530	TGFBR1	MIRT734034
UCA1	MIRT734797	KLF4	MIRT000308	TIMP2	MIRT732435
GLS2	MIRT734798	KLF5	MIRT000306	PTPRO	MIRT734853
		KREMEN1	MIRT021532	PPP2R2A	MIRT734854
		LYPLA2	MIRT021526	DAPK3	MIRT735333
		MAP2K6	MIRT021513		
		MCM2	MIRT732469		
		MDM2	MIRT007257		
		MEST	MIRT021508		
		MIXL1	MIRT021535		
		MMP1	MIRT021518		
		MMP12	MIRT021521		
		MMP14	MIRT021533		
		MSH3	MIRT734568		
		MTDH	MIRT733264		
		MTMR14	MIRT021522		
		MUC1	MIRT000426		
		**MYC**	MIRT004290		
		MYO5A	MIRT035535		
		MYO6	MIRT000457		
		MYOCD	MIRT733241		
		NAIP	MIRT731786		
		NANOG	MIRT035522		
		NDRG2	MIRT021510		
		NDUFA4	MIRT021524		
		NEDD9	MIRT006889		
		NFATC1	MIRT438543		
		NIPSNAP1	MIRT021531		
		NRAS	MIRT007107		
		NUDT1	MIRT006788		
		PAK4	MIRT006899		
		PARP8	MIRT000732		

	PIGF	MIRT021528
	PODXL	MIRT021537
	POU5F1	MIRT004904
	PPP3CA	MIRT000677
	PTP4A2	MIRT021519
	PXN	MIRT734440
	ROBO2	MIRT005809
	ROCK1	MIRT438282
	RPA1	MIRT732468
	**RPS6KB1**	MIRT438496
	RREB1	MIRT053081
	RTKN	MIRT021499
	SENP1	MIRT731217
	SERINC5	MIRT021507
	SERPINE1	MIRT006317
	SET	MIRT732467
	SMAD2	MIRT735099
	SMAD3	MIRT053650
	SOCS7	MIRT007248
	SOX2	MIRT000307
	SOX9	MIRT053648
	SP1	MIRT053757
	SP7	MIRT438643
	SPTBN1	MIRT731094
	SPTLC1	MIRT732470
	SRGAP1	MIRT005810
	STAT1	MIRT000626
	SWAP70	MIRT006747
	TGFB2	MIRT735095
	TGFBR2	MIRT053652
	TIRAP	MIRT004748
	TMEM9B	MIRT021520
	TNFSF13	MIRT054274
	TPM3	MIRT021512
	TPRG1	MIRT021515
	TSPAN6	MIRT021536
	TUG1	MIRT733664
	**VEGFA**	MIRT006215
	VPS51	MIRT735489
	YES1	MIRT000627

Bold targets are the ones shared by at least two microRNAs.
